# Variation in Volatile Organic Compounds in native, synanthropic accessions and cultivars of the musk strawberry (*Fragaria moschata* Duchesne ex Weston)

**DOI:** 10.1371/journal.pone.0289468

**Published:** 2023-08-10

**Authors:** Christiane M. Ritz, Detlef Ulrich, Sebastian Buschmann, Klaus Olbricht

**Affiliations:** 1 Chair of Biodiversity of Higher Plants, International, Institute (IHI) Zittau, Technical University Dresden, Dresden, Germany; 2 Department of Botany, Senckenberg Museum for Natural History Görlitz, Senckenberg – Member of the Leibniz Association, Görlitz, Germany; 3 Julius Kühn-Institute (JKI), Federal Research Centre for Cultivated Plants, Institute for Ecological Chemistry, Plant Analysis and Stored Product Protection, Quedlinburg, Germany; 4 Institut für Botanik, Technische Universität Dresden, Dresden, Germany; 5 Hansabred GmbH & Co. KG, Dresden, Germany; 6 Albrecht Daniel Thaer-Institute of Agricultural and Horticultural Sciences, Humboldt-Universität Berlin, Berlin, Germany; Institute for Biological Research, University of Belgrade, SERBIA

## Abstract

Prior to the world-wide dominance of *F*. *×ananassa* in strawberry production, native species had been cultivated in European gardens for centuries. Especially the musk strawberry (*F*. *moschata*) had been highly appreciated due to its fruit size and extraordinary aroma. Detailed studies on the diversity of the species’ fruit aroma are lacking, although breeding aims to improve strawberry aroma by complex crossings during recent years. Today a few cultivars, abandoned synanthropic occurrences and native populations of this species exist in Germany. Here we characterised aroma profiles of *F*. *moschata* accessions by analysing Volatile Organic Compounds. In particular, differences between native and cultivated accessions as well as the diversity in aroma profiles of native populations were investigated. Profiles of Volatile Organic Compounds were analysed by immersion stir bar sorptive extraction-gas chromatography-quadrupol mass spectrometry (imm-SBSE-GC-qMS). These data were compared with a genetic characterisation of samples based on eight microsatellite loci using univariate and multivariate statistical analyses. High amounts of furanones and the key compound methyl anthranilate were characteristic for the aroma profile of *F*. *moschata*. We detected a considerable diversity of Volatile Organic Compounds among accessions of *F*. *moschata*, particularly among genetically distinct samples from the same population. Native accessions contained more terpenoids but less esters and were moderately differentiated from cultivated samples. The observed patterns of Volatile Organic Compounds indicate that cultivated accessions had been selected for favourable aroma profiles and thus showing traces of domestication. Moreover, native populations harbour a great diversity of Volatile Organic Compounds, which could be also considered for future breeding efforts.

## Introduction

Strawberries (*Fragaria* L.) represent the most important berry crop world-wide [1]. Nowadays their industrial production nearly exclusively relies on cultivars of the octoploid *F*. *×ananassa* (Duchesne ex Weston) Duchesne ex Rozier, which originated by accidental hybridisation between the American species *F*. *virginiana* Mill. and *F*. *chiloensis* (L.) Mill. However, before the advent of the profitable production of this large-fruited hybrid, native species had been cultivated in Europe for several hundred years until the 19^th^ century [2]. Central Europe harbours three native strawberry species: the diploid woodland strawberry (*F*. *vesca* L., 2n = *2x* = 14), the diploid green strawberry (*F*. *viridis* Weston, 2n = *2x* = 14) and the hexaploid musk strawberry (*F*. *moschata* Duchesne ex Weston, 2n = *6x* = 42). The latter had been widely cultivated for its very intensive sweet-floral aroma and larger fruits compared to diploids, resulting in a number of cultivars. However, the rather complicated growing conditions due to the species’ dioecy and its poor shelf life led to a nearly complete abandonment of cultivation, except in Northern Italy at more regional level [2, 3]. Additionally, the successful cross-breeding of *F*. ×*ananassa* resulted in large assortments with highly productive cultivars and displaced all selections based on native European species.

Recent studies of native and synanthropic populations of *F*. *moschata* in Germany revealed genetically highly diverse native populations in Saxony and Bavaria, while all other occurrences of *F*. *moschata*, especially in northern and western Germany, were of synanthropic origin and consisted of a few but widely distributed female clones [4, 5]. Remarkably, synanthropic populations showed no close relationship to nearby native stands, but they were − in line with their distribution in Germany − either related to northern cultivars from France and England (e.g. ’Hautbois’, ’Capron Royal’) or to southern cultivars from Italy (e.g. ’Profumata di Tortona’; [5]). The high genetic diversity of native populations is presumably caused by the species’ obligate outcrossing due to the above mentioned dioecy and its proposed allopolyploid origin [6–10].

The characteristic strawberry flavour is, besides the amount and ratio of sugars and acids, mainly influenced by Volatile Organic Compounds (VOC). Such VOC are shown to be highly diverse in strawberries and include among others esters, alcohols, aldehydes, ketones, furanones, and terpenoids [11]. Volatile organic compounds do not only create fruit flavour but play also a role in the defence against diseases and herbivores [12–14]. More than 970 VOC have been reported in strawberry fruits in total, however, 67% of these substances were found only once [15]. Moreover, it has been shown that only a small share of VOC (“key compounds”) influences aroma impression [16–18]. As strawberry breeding focused mainly on yield, visual impression and shelf life, flavour was largely neglected resulting in domestication effects expressed in a pronounced VOC depletion and thus poor aroma quality in modern cultivars compared to older ones and wild strawberry species [19, 20]. Ulrich et al. (1997) classified *F*. *×ananassa* cultivars into aroma types mainly based on the presence of methyl anthranilate and other short chain esters [20]. Methyl anthranilate is the key VOC characterising the intensive aroma of *F*. *vesca* and is found, in example, in the highly appreciated old German cultivar ’Mieze Schindler’, whereas it is absent in modern cultivars [21]. In general, most VOC studies focused on *F*. *×ananassa*, yet only a limited number of wild strawberry species has been investigated [3, 21–25] and, in particular, intraspecific variation has been hardly considered [21]. In *F*. *moschata* data exist for the cultivars ’Profumata di Tortona’ and ’Capron Royal’ [3, 26], but rather anecdotal data are available for native populations [22, 24]. These studies demonstrated that esters were the predominant class of substances and among these, *F*. *moschata* contained considerable amounts of methyl anthranilate [22, 26]. In addition, the furanone mesifurane, responsible for a caramel-like note, was substantially higher in *F*. *moschata* compared to *F*. *vesca* [3, 22].

In this study we aimed to compare VOC profiles between cultivars, synanthropic and native accessions of *F*. *moschata*. Particularly, we were interested whether VOC profiles are suited to characterise different accessions of *F*. *moschata*, i.e. to detect a domestication effect. Therefore, we performed a gas-chromatographic analysis (imm-SBSE-GC-qMS) on 56 accessions *F*. *moschata* from the germplasm collection “*Professor Staudt Collection*” [27]. In addition, we wanted to study the diversity of VOC profiles across various native accessions and investigate whether genetic relatedness (estimated by microsatellite analyses) is correlated with distances calculated from VOC profiles.

## Material and methods

### Plant material and fruit harvest

Eleven cultivars, 28 synanthropic accessions from six sites (five sites from Germany, one from the Netherlands) and two native stands of *F*. *moschata* from Saxony (Germany) maintained in the germplasm collection “*Professor Staudt Collection*” [27] hosted by Hansabred (S1 Table) were analysed. *Fragaria moschata* is not protected by European or German law, and all accessions from “*Professor Staudt Collection*” were sampled prior to 2014, cultivars are commercially available and all other samples were originally collected in Germany (with the exception of one accession from The Netherlands), so the use of this material is not restricted by the Nagoya protocol. In addition, 14 living specimens collected in 2014 from the native site Ziegenbusch (Niederau, Saxony, Germany) were added to the germplasm collection and analysed here. The conservation agency *Untere Naturschutzbehörde des Landkreises Meißen* (permit Number: 20404/364.21-NSg D29 Vorgänge/OWiG#33-15805/2017) and to the landowner *Landesverein Sächsischer Heimatschutz* permitted access and sampling of *F*. *moschata* at the protected area Ziegenbusch. In 2015 all plants were propagated by runners and grown in two clay pots (20 cm diameter) in “substrate 5” of Klasmann company (https://klasmann-deilmann.com/) with at least eight plants in total. During winter plants were maintained in a non-heated plastic tunnel and forced in cultivation in spring 2016. After flowering and pollination supported by bumblebees, fruit harvest started at 23rd of May and ended at 21st of June 2016. Available fruits ranged from 27 to 283 fruits per accession with total sample weights from 41.6 g to 380 g and an average single fruit weight between 0.68 g to 2.42 g. All typical and healthy fruits were immediately frozen at -20 °C and used for later analysis as a batch sample.

### Analysis of organic volatile compounds (VOC) by imm-SBSE-GC-qMS

To prepare an enzyme inhibited strawberry juice, all frozen fruits without sepals were homogenized in one volume part of a solution of 18.6% (m/v) NaCl using a household mixer (Bosch professional MSM 71) for 2 min. The homogenate was centrifuged 4000 rpm for 30 min. One hundred millilitre of the supernatant were mixed with 10 μl internal standard (0.1% (v/v) 2,6-dimethyl-5-hepten-2-ol dissolved in ethanol). For each sample, three head-space vials containing 3 g NaCl each for salt-saturation were filled with 10 ml of the supernatant, sealed with magnetic crimp caps including septum, and stored at 4 °C.

An aliquot of 8 ml of the saturated homogenate but without the solid NaCl deposit was transferred in an empty glass vial for volatile isolation by immersion SBSE. A stir bar with 0.5 mm film thickness and 10 mm length coated with polydimethysiloxane (PDMS) was placed in the liquid (Gerstel, Mülheim an der Ruhr, Germany). The stir bar was moved at 350 rpm at room temperature for 45 min. After removal from the strawberry juice, the stir bar was rinsed with purified water, gently dried with a lint-free tissue and then transferred into a glass tube for thermal desorption and subsequent GC analysis.

Parameters for the thermal desorption unit (TDU, Gerstel) and the cold injection system (CIS4, Gerstel) were the following: thermal desorption at 250 °C, cryo-trapping at -150 °C. The TDU-CIS4 unit was used in Gerstel-modus 3: TDU splitless and CIS4 with 15 ml/min split flow. The analyses were performed with an Agilent Technologies 6890N gas chromatograph equipped with an Agilent 5975B quadrupol MS detector (Agilent, Waldbronn, Germany). Compounds were separated on a polar column Zebron ZB-Wax Plus 30 m length × 0.25 mm inner diameter × 0.5 μm film thickness. Helium was used as a carrier gas with a column flow rate of 1.1 ml/min. Temperature program was: 45 ºC (3 min), followed by a temperature gradient of 3 K/min to 210 ºC for 30 min. The mass spectrometer was used with electron ionisation at 70 keV in the full scan mode. All samples were run with two analytical replicates from an identical part of the same supernatant, subsequently, means were calculated.

The software ChromStat2.6 (Analyt, Müllheim, Germany) was used for data processing [28]. Data inputs for ChromStat2.6 were raw data from the TIC (total ion chromatogram) percentage reports (retention time/peak area data pairs) performed with the software package Chemstation (v. Rev.B.02.01.-SR1) by Agilent. Using ChromStat2.6, the chromatograms were divided in up to 200 time intervals, each of which represented a peak (substance) occurring in at least one chromatogram of the analysis set. The peak detection threshold was set on the 10-fold value of noise. The values are given as raw data (peak area in counts, S2 Table) and were then normalised by setting the sum of the raw data of all VOC per sample to 1.0, representing a semi-quantification for further statistical analyses. The method applied here by simultaneously detecting a large number of VOC per sample allows only for a semi-quantification and not for presenting exact units of concentration (quantitative data) as reviewed by Ulrich et al. 2018 [15].

### Genetic characterization

Genetic fingerprints obtained from eight microsatellite loci were generated in our previous studies: Data from synanthropic accessions, accessions from Upper Lusatia and cultivars were taken from [5] and fingerprints from native accessions in Germany were originally obtained by S. Buschmann in his unpublished master thesis [29], and these data were newly combined for this analysis. The microsatellite marker *ChFaM1* was taken from Gil-Ariza et al. (2006) [30]. Microsatellite markers *FG2cd*, *FG7ef*, *FG1ab*, *UFFa3-D11ab*, *FG2ab*, *FG1cd* and *FG7ab* were originally published in Chambers et al. 2013 [31]. The locus *UFFa3-D11ab* was newly amplified from synanthropic accessions and cultivars for the present study. The data set of 56 accessions contained 46 alleles, which were analysed as allelic phenotypes: presence of an allele was recorded but copy number was not identified because genotypes could not be determined in hexaploid samples. Individuals exhibiting the same allelic composition across all eight loci were assigned as clones resulting in 23 clone groups (S3 Table).

### Statistical analyses

All measurements of VOC (see original data in S2 Table) were transformed to proportions (the sum of all identified VOC per sample were set to 1.0; S4 Table). In addition, the 58 different VOC detected were summarized to 13 classes of substances (S5 Table). An overview about CAS registry numbers and substance classification is given in S6 Table. In order to classify samples according to their VOC profiles, we transformed data using all accessions (including clones) into Euclidean distances and performed a Principal Component Analysis based on centred and standardized data with the *R* package *vegan* [32] and presented the results as a biplot using *ggplot2* [33] in *R* environment [34]. Based on high loadings of the PCA we selected single VOC for univariate analyses (not shown). Therefore, we calculated mean values of norm% per clone group and tested for differences in single VOC between cultivars and synanthropic accessions on one hand and native accessions on the other by two-tailed t-tests. We presented significant results as Box-Whisker-Plots with the software past v. 4.02 [35].

We used the *R* package *polysat* for statistical analysis of microsatellite data [36]. Data of present alleles were transformed in Bruvo distances [37] and a Principal Coordinate Analysis was subsequently performed (excluding clones) using *ggplot2*. In addition, we calculated a Mantel to test for correlation between genetic distance (Bruvo distance) and distance in VOC profiles (Euclidean distance) with *R* package *ade4* [38–40] based on 9999 permutations.

## Results

**Fig 1:**
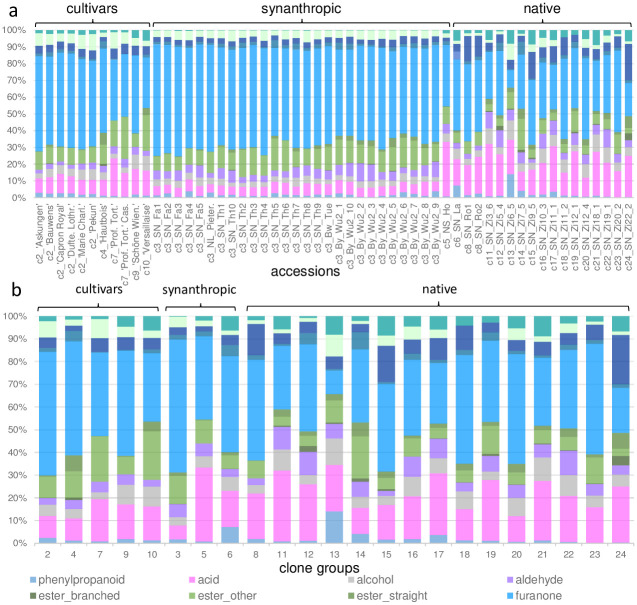
Proportions of VOC classes in cultivars, synanthropic and native accessions of *F*. *moschata*. a) all investigated accessions separately (clone groups are given as numbers above bars) and b) as means per clone group. Sample abbreviations are according to S1 Table, for VOC classes see S5 and S6 Tables.

We detected a considerable variation in quality and proportion of in total 58 VOC across different accessions of *F*. *moschata* (S4 and S5 Tables). Volatile Organic Compounds were summarised to 13 classes of substances and presented for each investigated accession including clones (Fig 1a) and as means per clone group (Fig 1b). The highest share of VOC was found for furanones, followed by acids. Samples of the same clone group were very similar with respect to classes of VOC, whereas genetically different samples from native populations, even within the same population, varied considerably (Fig 1a). In general, native accessions contained proportionally less “other esters” (t-test: T_7, 16_ = 3.64, p = 0.002) and more terpenoids (t-test: T_7, 16_ = 2.14, p = 0.04) compared to synanthropic accessions and cultivars.

**Fig 2:**
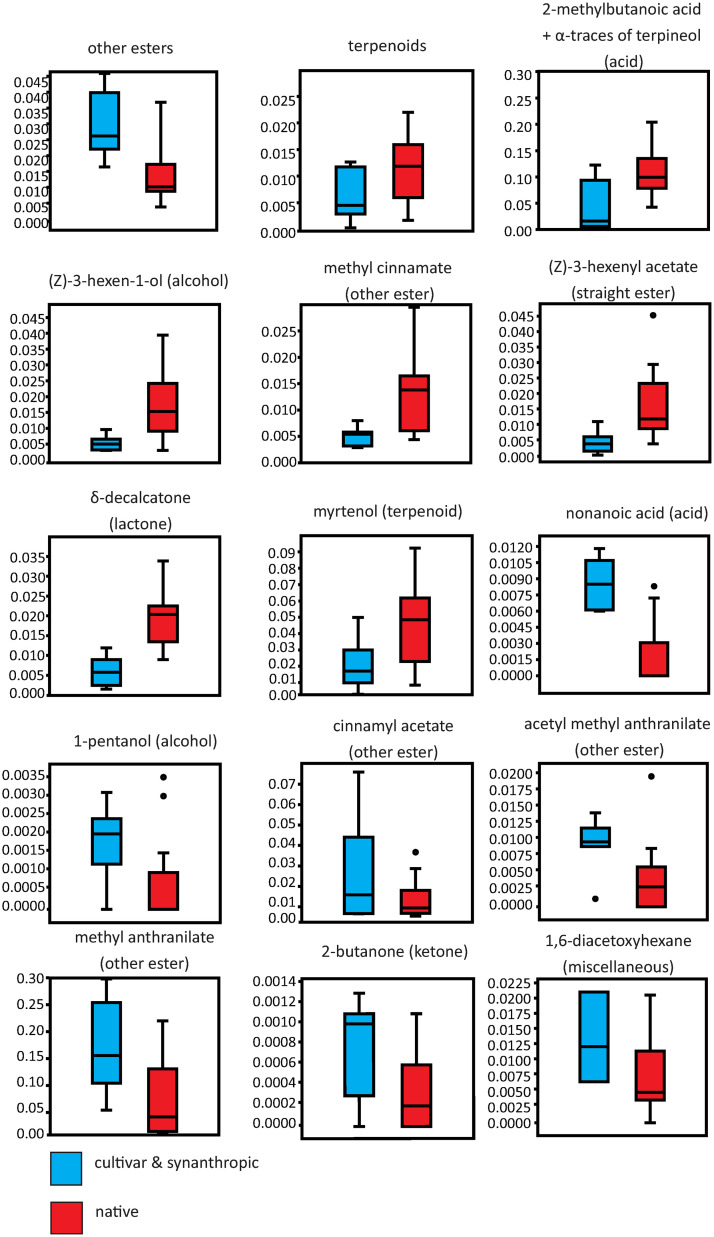
Box-Whisker plots for proportions of VOC. Volatile Organic Compounds are given as proportions (sum of all VOC per sample was set to 1.0). Volatile Organic Compounds were calculated from means per clone group, and only those are shown, which differed significantly between cultivars and synanthropic accessions on one hand (n = 7) and native samples (n = 16) on the other hand. The respective substance class for each VOC is given in brackets.

At the level of single VOC native accessions were richer in the acid 2-methyl butanoic acid (t-test: T_7, 16_ = 3.23, p = 0.004; unseparated from traces of α-terpineol), the alcohol (*Z*)-3-hexen-1-ol (t-test: T_7, 16_ = 2.98, p = 0.007), the “other ester” methyl cinnamate (t-test: T_7, 16_ = 29.50, p = 0.008), the straight ester (*Z*)-3-hexenyl acetate (t-test: T_7, 16_ = 2.84, p = 0.010), the lactone δ-decalactone (t-test: T_7, 16_ = 4.94, p < 0.001) and the terpenoid myrtenol (t-test: T_7, 16_ = 2.90, p = 0.025; Fig 2). In contrast, native accessions contained lower shares of nonanoic acid (t-test: T_7, 16_ = 5.86, p < 0.001), the alcohol 1-pentanol (t-test: T_7, 16_ = 2.49, p = 0.021), the “other esters” cinnamyl acetate (t-test: T_7, 16_ = 2.08, p < 0.050), acetyl methyl anthranilate (t-test: T_7, 16_ = 2.42, p = 0.025), and methyl anthranilate (t-test: T_7, 16_ = 2.92, p = 0.008), the ketone 2-butanone (t-test: T_7, 16_ = 2.24, p = 0.036), and the substance 1,6-diacetoxyhexane (t-test: T_7, 16_ = 2.08, p = 0.050; Fig 2). The furanone furaneol was nearly absent in the native accessions (S3 Table).

**Fig 3:**
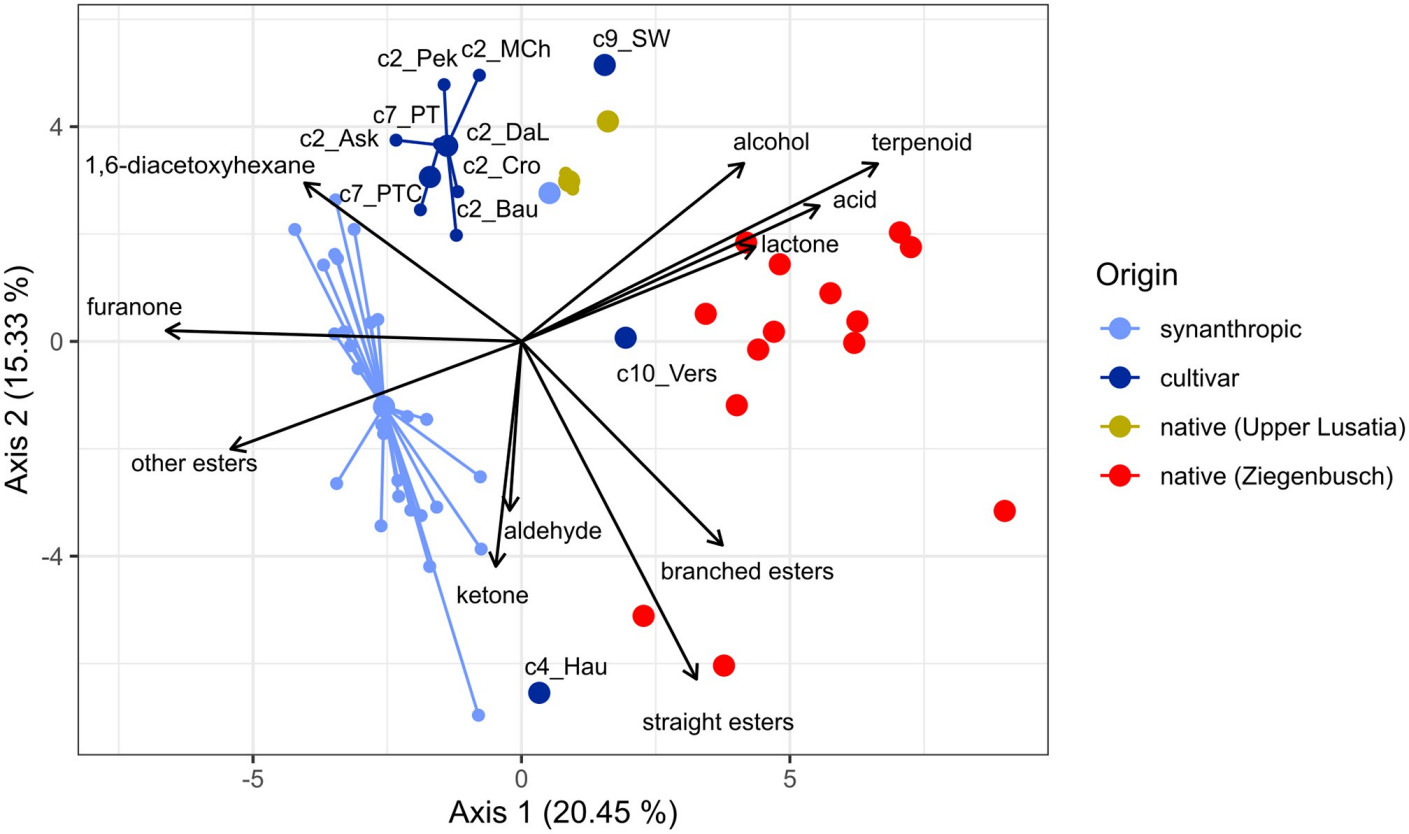
Biplot of the Principal Component Analysis based on 58 VOC of native + synanthropic samples and cultivars of *F*. *moschata*. Substance classes of VOC, which significantly correlated (p<0.05) with PC1 and PC2, were plotted (see S5 Table). Cultivars are presented in dark blue, synanthropic samples in light blue, native samples in green or red (Saxon accessions from Upper Lusatia and Ziegenbusch, respectively). The 24 clone groups determined by microsatellite analyses are indicated by lines connecting the single accessions (small dots) and the mean value for the clone group (big dots). Sample abbreviations are c2_Ask = c2_’Askungen’, c2_Bau = c2_’Bauwens’, c2_Cro = c2_’Capron_Royal’, c2_Pek = c2_’Pekun’, c2_MCh = c2_’Marie Charlotte’, c2_DaL = c2_’Dufterdbeere aus Lothringen’, c4_Hau = c4_’Hautbois’, c7_PT = c7_’Profumata di Tortona’, c7_PTC = c7_’Profumata di Tortona’ Casalini w, c9_SW = c9_’Schöne Wienerin’ and c10_Vers = c10_’Versaillaise’ according to S1 Table.

The Principal Component Analysis based on all detected VOC separated native samples from cultivars and synanthropic accessions along PC1 (Fig 3). The cultivar ’Versaillaise’ clustered closely to native samples. The cultivars ’Hautbois’ and ’Schöne Wienerin’ had an intermediate position along PC1, together with some synanthropic samples, but were separated from each other along PC2. Native accessions contained higher levels of alcohols, terpenoids, acids and lactones, whereas cultivars and synanthropic samples were rich in furanones, 1,6-diacetoxyhexane and “other esters”. Samples originating from the same clone group were mostly in close proximity to each other but did not have identical VOC profiles. In contrast, native accessions from the Saxonian site Ziegenbusch belonged to different genotypes and were largely scattered across the right side of the PCA plot.

**Fig 4:**
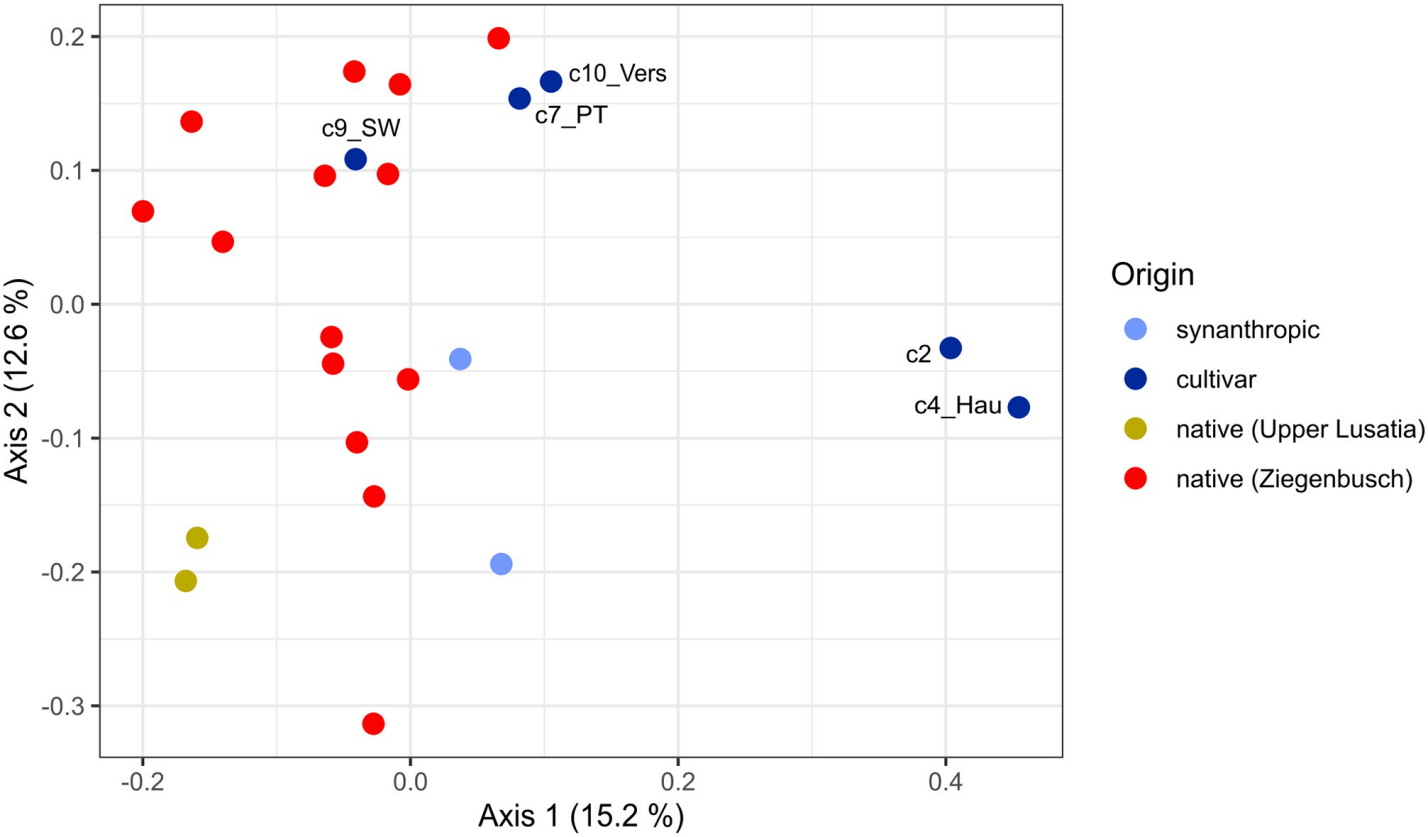
Principal Coordinate Analyses based on Bruvo distances obtained from eight microsatellite alleles. Clones were only included once. Cultivars are presented in dark blue, synanthropic samples in light blue, native samples in green or red (Saxon accessions from Upper Lusatia and Ziegenbusch, respectively). Sample abbreviations are c2 = clone group 2 (six cultivars), c4_Hau = c4_’Hautbois’, c7_PT = clone group 7 (two accessions of ‘Profumata di Tortona’), c9_SW = c9_’Schöne Wienerin’, c10_Vers = c10_’Versaillaise’ according to S1 Table.

The Principal Coordinate Analysis based on genetic data (clones were only represented once, S6 Table) revealed a slight differentiation between native accessions, which were clustered on the left side, whereas synanthropic samples and cultivars were distributed in the centre and on the right side of the plot (Fig 4). The cultivars of the clone group 2 (c2; six cultivars) and the cultivar ’Hautbois’ were thereby most clearly separated along the first axis. Samples from the site Ziegenbusch were widely scattered along the second axis.

Correlation between genetic distances (Bruvo distance) and Euclidean distances obtained from 58 VOC showed no significant result (Mantel test: r = 0.17, p = 0.06).

## Discussion

In this study we showed that cultivars and synanthropic accessions *F*. *moschata* differed from native accessions according to their aroma profiles. In addition, we found that different genotypes from native populations were characterised by highly variable compositions of VOC.

### VOC content of *F*. *moschata*

In total, we semi-quantified 58 VOC in *F*. *moschata*, which were considerably less in number compared to results of Negri et al. 2015 (131 VOC) and Pet’ka et al. 2012 (100 VOC; [3, 22]). This might be explained by methodological differences, i.e. different sample preparation methods and analysis methods, i.e. gas chromatography versus imm-SBSE-GC-qMS used here (see [15]). Differences between the volatile profile have also been observed in *F*. *chiloensis* using extraction methods ([23]; DOI 10.1002/jsfa.6412) and with the intact fruit ([41]; https://doi.org/10.1021/jf901693j). According to substance classes we detected mainly furanones, esters and acids (Fig 1). Furanones, with caramel-like odour [11] were highly abundant in *F*. *moschata*, and among these we detected a high proportion of mesifurane (S4 Table). In contrast, furaneol was nearly absent, which is in accordance to previous analyses [24]. High shares of mesifurane appear to be a characteristic feature of *F*. *moschata* aroma because also Negri et al. (2015) observed a 900 fold increase of this substance in comparison to *F*. *vesca* [3]. However, the proportion of furanones varied considerably between accessions (S3 Table, Fig 1b): although differences were not significant between cultivated and native samples, some native samples (clone group 13, 24) contained very low shares. Moreover, furaneol was completely absent in native accessions from the site Ziegenbusch (S4 Table). In general, esters were reported to be the most abundant compounds in strawberries [15] and in *F*. *moschata* [3]. Methyl anthranilate was found also in all samples, although higher proportions were detected in cultivated accessions (Fig 2, see below). This ester is responsible for the characteristic intensive sweet aromatic impression of wild strawberries, especially in *F*. *vesca*, but appeared to be absent in most modern strawberry cultivars [20]. Our results are in agreement with previous publications in which ’Profumata di Tortona’ contained higher shares of methyl anthranilate compared to *F*. *vesca* [3, 26], while native accessions of *F*. *moschata* were less rich in methyl anthranilate compared to *F*. *vesca* [24]. Accordingly, we confirmed the findings of Negri et al. (2015) and Ulrich et al. (2007) that the terpenoid linalool with a pleasant floral odour plays a much less important role for flavour in ’Profumata di Tortona’ compared to *F*. *vesca* and *F*. *×ananassa* [3, 24].

#### Differences between cultivated and native accessions of *F*. *moschata*

Both, multivariate VOC analyses (Fig 3) and population genetic data (Fig 4; [5]) moderately differentiated between native and cultivated accessions of *F*. *moschata*. Remarkably, VOC analyses showed a closer relationship of the cultivars ’Versaillaise’ and ’Hautbois’ to native accessions (Fig 3), while the genetic analyses revealed that ’Versaillaise’, ’Profumata di Tortona’ and ’Schöne Wienerin’ were close to native accessions (Fig 4), which was partly confirmed by a more detailed population genetic study [5]. Therein, ’Versaillaise’ and ’Schöne Wienerin’ were closely grouped to native populations, but these accessions were also part of a northerly distributed synanthropic cluster, while ’Profumata di Tortona’ belonged to a cluster of more southerly distributed synanthropic occurrences in Germany. However, pedigrees of these cultivars are hardly known, but at least for ’Schöne Wienerin’ there are references to the breeder Gottlieb Goeschke (1874), who introduced his new cultivar as a selection in 1889, exist: He and his father worked in Koethen (Germany) and it is documented that they also used native accessions in their breeding activities [42]. The morphological characteristics of ’Schöne Wienerin’ (fruit size and shape etc.) resembles native accessions from the Saxon population Ziegenbusch [43].

Native accessions contained lower proportions of “other esters” but a larger share of terpenoids compared to cultivated accessions. This is in accordance to expectations by domestication, because esters are the key compounds of pleasant strawberry flavour [11, 15], so cultivars might have been selected for the presence of short esters, as is the case for *F*. *×ananassa* [21]. As mentioned above, methyl anthranilate characterises the sweet, floral aroma of wild strawberries, and again we found a smaller share of this VOC in native accessions of *F*. *moschata* (Fig 2). However, this pattern did not hold for all esters, i.e., here we found higher amounts of some esters in cultivated samples but lower amounts for others (Fig 2).

A similar pattern arose for terpenoids, cultivars and synanthropic accessions were characterized by lower shares of myrtenol compared to native accessions (Fig 2), corresponding to the complete loss of myrtenol in cultivars of *F*. *×ananassa* [44]. In general, a higher share of terpenoids in wild species has been also observed in *F*. *vesca* in comparison to *F*. *×ananassa* [21]. The presence of terpenoids correlated with consumer acceptance in *F*. *×ananassa* when the amount of lactones was simultaneously high [45], but high concentrations of terpenoids with at the same time missing lactones negatively influenced acceptance because of their harsh turpentine-like, woody aroma. In contrast, low amounts of terpenoids due to selective breeding might cause higher susceptibility to fungal diseases, since an upregulation of terpenoid metabolism is discussed to be responsible for the resistance of *F*. *nilgerrensis* Schltdl. ex J. Gay to *Colletotrichum* infections [46].

Lactones were also detected in all accessions (Fig 1), but we found higher proportions, especially for δ-decalactone (Figs 2 and 3) in native compared to synanthropic accessions and cultivars. The low amount of lactones in the latter is in contrast to many modern *F*. *×ananassa* cultivars, in which an excess of lactones is responsible for a predominant peach note [18], but lactones also enhance the intensity of sweetness [17].

Even though we found different patterns in the selection process between *F*. *moschata* and *F*. *×ananassa* with respect to VOC, the higher proportions of furanones and “other esters”, especially of methyl anthranilate, in cultivated and synanthropic samples in comparison to wild accessions can be interpreted as a parallel domestication effect by which aroma profiles with higher acceptance were selected.

### Implications for breeding

Although we detected a moderate differentiation between native and synanthropic samples by both, VOC (Fig 3) and genetic data (Fig 4), Mantel tests did not reveal significant correlations between the two distance measures. However, genetically identical accessions showed very similar VOC profiles with respect to substance classes (Fig 1a) and were closely grouped but not identical in the multivariate analyses based on all VOC (Fig 3). Microsatellites are neutral genetic markers, and VOC profiles are controlled by various complicated biochemical pathways under selection [47], so a direct correlation between distances is not unexpected. Our study showed that simple and cost-effective microsatellites markers are still a very valuable tool to characterize accessions from germ plasm collections and are suited to accompany breeding efforts. Genetic characterisation is extremely helpful for choosing appropriate cross-breeding partners, as our data showed a considerable variation in VOC between genetically different samples. We detected an amazing variation of VOC in native populations, even among (genetically different) accessions from the same site (Ziegenbusch), while synanthropic accessions and cultivars resembled each other in terms of substance classes and individual VOC (Fig 1). Variation of VOC profiles within a species /population has hardly been studied, with only anecdotal data for some native accessions of *F*. *vesca* and *F*. *moschata* [21, 22]. The here observed great diversity of VOC patterns corresponds to the great variation in fruit morphology reported from the same accessions [43, 48]. Thus, our study provides evidence for the first time of intra-population VOC diversity, which might be worth for a more substantial screening in breeding efforts. Although VOC analyses were performed using plants grown under horticultural conditions, numerous studies have shown that factors such as season, plant rigour, harvest date and storage conditions [11, 15] influence the expression of aroma compounds. Therefore, the variation of VOC profiles in natural strawberry populations over several years would be also an interesting topic for future research.

In general, the richness of VOC and the particular flavour in *F*. *moschata* is promising for breeding attempts. The differences in the VOC related domestication effects between *F*. *×ananassa* [49] and *F*. *moschata* suggests that the potential for intraspecific breeding is scarcely used in *F*. *moschata*. There is no current important breeding activity on the hexaploid species level worldwide, partly because limitations in fruit size and firmness of fruits are expected. Therefore, introgression with the cultivated strawberry *F*. *×ananassa* seams reasonable. However, there are substantial difficulties in interspecific hybridisation due to ploidy differences and other incompatibilities [50]. Pre-breeding experiments next to already published synthetic polyploids [51] are necessary first.

## Supporting information

S1 Table Origin of sampled material. All accessions were taken from the germ plasm collection “Professor Staudt Collection” [27] hosted by Hansabred.
(XLSX)

S2 Table Original data of GC analysis resulting in 58 VOC detected across 56 accessions of *Fragaria moschata*. VOC are ordered according to Retention Time (mean across two technical replicates) given above each VOC detected.
(XLSX)

S3 Table Presence/absence data of 46 alleles from eight microsatellite markers across 56 accessions of *Fragaria moschata*. (XLSX)

S4 Table Normalized data of 58 VOC detected across 56 accessions of *Fragaria moschata*. The sum of all VOC per sample were set to 1.0 and proportions are presented in coloured cell as heat map (green low percentages, red high percentages). VOC are ordered according to substance classes.
(XLSX)

S5 Table Means of proportions per 13 class of substances detected across 56 accessions of *Fragaria moschata*. Proportions are presented in coloured cell as heat map (green low proportion, red high proportion).
(XLSX)

S6 Table Overview about Retention Times, substance classification, and CAS registry numbers. Retention Times are given as means across two technical replicates.
(XLSX)
